# Phantom-based analysis of variations in automatic exposure control across three mammography systems: implications for radiation dose and image quality in mammography, DBT, and CEM

**DOI:** 10.1186/s41747-024-00447-z

**Published:** 2024-04-16

**Authors:** Gisella Gennaro, Sara Del Genio, Giuseppe Manco, Francesca Caumo

**Affiliations:** 1 Veneto Institute of Oncology (IOV), IRCCS, Via Gattamelata 64, Padua, 35128 Italy; 2 AORN A. Cardarelli, Naples, Italy

**Keywords:** Automatic exposure control, Mammography, Radiation dosage, Radiographic image enhancement, Phantoms (imaging)

## Abstract

**Background:**

Automatic exposure control (AEC) plays a crucial role in mammography by determining the exposure conditions needed to achieve specific image quality based on the absorption characteristics of compressed breasts. This study aimed to characterize the behavior of AEC for digital mammography (DM), digital breast tomosynthesis (DBT), and low-energy (LE) and high-energy (HE) acquisitions used in contrast-enhanced mammography (CEM) for three mammography systems from two manufacturers.

**Methods:**

Using phantoms simulating various breast thicknesses, 363 studies were acquired using all available AEC modes 165 DM, 132 DBT, and 66 LE-CEM and HE-CEM. AEC behaviors were compared across systems and modalities to assess the impact of different technical components and manufacturers’ strategies on the resulting mean glandular doses (MGDs) and image quality metrics such as contrast-to-noise ratio (CNR).

**Results:**

For all systems and modalities, AEC increased MGD for increasing phantom thicknesses and decreased CNR. The median MGD values (interquartile ranges) were 1.135 mGy (0.772–1.668) for DM, 1.257 mGy (0.971–1.863) for DBT, 1.280 mGy (0.937–1.878) for LE-CEM, and 0.630 mGy (0.397–0.713) for HE-CEM. Medians CNRs were 14.2 (7.8–20.2) for DM, 4.91 (2.58–7.20) for a single projection in DBT, 11.9 (8.0–18.2) for LE-CEM, and 5.2 (3.6–9.2) for HE-CEM. AECs showed high repeatability, with variations lower than 5% for all modes in DM, DBT, and CEM.

**Conclusions:**

The study revealed substantial differences in AEC behavior between systems, modalities, and AEC modes, influenced by technical components and manufacturers’ strategies, with potential implications in radiation dose and image quality in clinical settings.

**Relevance statement:**

The study emphasized the central role of automatic exposure control in DM, DBT, and CEM acquisitions and the great variability in dose and image quality among manufacturers and between modalities. Caution is needed when generalizing conclusions about differences across mammography modalities.

**Key points:**

• AEC plays a crucial role in DM, DBT, and CEM.

• AEC determines the “optimal” exposure conditions needed to achieve specific image quality.

• The study revealed substantial differences in AEC behavior, influenced by differences in technical components and strategies.

**Graphical Abstract:**

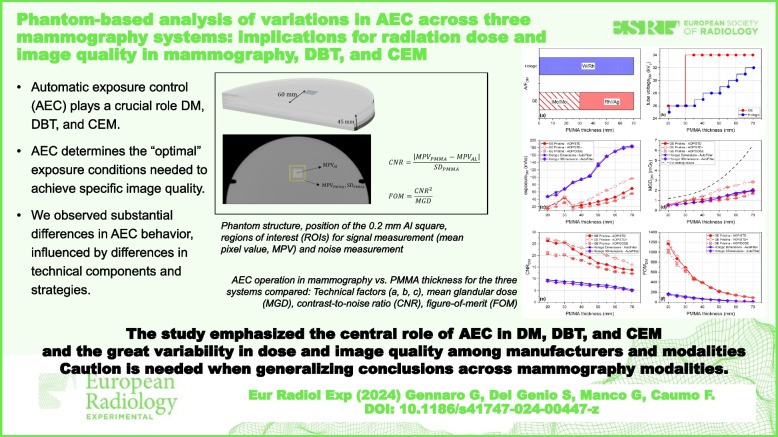

## Background

The role of automatic exposure control (AEC) in mammography systems is crucial, as it determines the “optimal” exposure conditions needed to achieve specific image quality (IQ) based on the unique absorption characteristics of compressed breast tissue. In previous screen/film mammography, a dose detector placed beyond the screen-film cassette interrupted the x-ray beam when it accumulated a threshold dose corresponding to the desired optical density for a particular type of film [1, 2]. However, the advent of digital mammography (DM) in the early 2000s led to a complete redesign of AECs.

DM systems now use the detector for both exposure control and image acquisition. In particular, systems employing a flat panel detector [3] perform a brief preliminary exposure (lasting a few milliseconds) to assess the overall breast absorption measuring the height of the compression paddle as an estimate of breast thickness, and identify the most absorbent area. The choice of anode/filter (A/F) material and x-ray voltage for image acquisition depends on the breast absorption and IQ index used by the imaging system manufacturer designing the AEC design. Meanwhile, the exposure value (tube current × exposure time, mAs) is determined according to the area of highest absorption [4, 5]. DM, by separating image acquisition from postprocessing and displaying, has facilitated the transition of the AEC operation from a fixed dose of radiation at the detector level to a focus on achieving “target IQ” [6, 7].

In addition, DM has evolved with digital breast tomosynthesis (DBT), a pseudo-three-dimensional method reconstructing images of breast slices from limited low-dose projections acquired at various angles [8, 9]. Contrast-enhanced mammography (CEM), which uses a dual-energy technique after the intravenous administration of iodinated contrast agent, is another DM advance that allows to obtain both standard and contrast-enhanced breast images at the same time [10]. AEC automatically determines the technical factors for each DBT projection by dividing the total exposure value by the number of projections and separates the two x-ray spectra below and above the iodine absorption peak to present the low-energy (LE) images substantially equivalent to standard mammograms and recombined LE-high-energy (HE) images as only contrast images in CEM.

In this phantom study, our focus was on examining the variability in AEC behavior across three distinct mammography systems, for each of the three imaging modalities available (DM, DBT, and CEM) and its impact on the optimization of IQ *versus* dose. In particular, we sought to understand how the choices made by each manufacturer, which include system components and AEC designs, contribute to the balance between IQ and radiation dose.

## Methods

### Imaging systems

The comparison was performed between three systems, a GE Senographe Pristina (GE Healthcare, Chicago, IL, USA) and two Hologic units, Selenia Dimensions and Selenia 3Dimensions, respectively (Hologic, Bedford, MA, USA), for all the modalities (DM, DBT, and CEM) and all the AEC modes available on each unit. There are many differences in system design, including x-ray source (anode and filter materials), detector technology, and tomosynthesis protocol (tube motion, number of projections, angle, geometry, presence/absence of anti-scatter grid). Table 1 summarizes the main physical characteristics of the three systems.

**Table 1:** Main physical characteristics of the three mammography systems compared

System	Senographe Pristina	Selenia Dimensions	Selenia 3Dimensions
Manufacturer	GEHC	Hologic	Hologic
Detector type	CsI FPD	a-Se FPD	a-Se FPD
Pixel pitch (μm)	100	70 (2 × 2 binning in DBT)	70
Modalities	DM DBT CEM	DM DBT	DM DBT CEM
Anode(s)	Mo and Rh	W	W
Filter(s)	Mo, Ag, Cu^a^	Rh, Ag, Al^b^, Cu^a^	Rh, Ag, Al^b^, Cu^a^
DBT protocol			
Tube motion	Step-and-shoot	Continuous	Continuous
Projections	9	15	15
Angle	25° (± 12.5°)	15° (± 7.5°)	15° (± 7.5°)
Geometry	Partial isocentric	Full isocentric	Full isocentric
Scatter	Grid	No Grid	No Grid
N° AEC modes	DM: 3 (AOP/STD, AOP/STD+ , AOP/DOSE) DBT: 2 (AOP/STD, AOP/STD+) CEM: 1 (AOP/STD)	1 (AutoFilter)	1 (AutoFilter)

AOP/STD, AOP/STD+ , AOP/DOSE, AutoFilter are the names given by the two manufacturers to their AEC modes

*FPD* Flat panel detector, *CsI* Cesium iodine, *a-Se* Amorphous selenium, *DM* Digital mammography, *DBT* Digital breast tomosynthesis, *CEM* Contrast-enhanced mammography, *AEC* Automatic exposure control

^a^Cu filter is used by both systems (GE Pristina and Hologic 3Dimensions) for the only high-energy image in CEM mode

^b^Al filter is used by Hologic systems in DBT mode

The GE Pristina combines a dual track x-ray tube (molybdenum, Mo, and rhodium, Rh) with a scintillator-based (cesium iodide, CsI) flat panel detector (FPD) 100-μm pixel pitch, while both Selenia systems use a single track (tungsten, W) x-ray tube with a photoconductor-based (amorphous selenium, a-Se) FPD 70-μm pixel pitch. The GE Pristina performs DBT with 9 projections at 25° in partial isocentric geometry, with step-and-shoot tube motion, maintaining the antiscatter grid used in DM. Otherwise, DBT by the two Hologic units is performed with continuous tube motion, acquiring 15 projections at 15° in full isocentric geometry, without an antiscatter grid. The only difference between the two Hologic systems is that while in DBT modality the Dimensions averages the signal from 2 × 2 adjacent pixels (binning) to increase the signal-to-noise ratio (while reducing spatial resolution), the 3Dimensions system works in full resolution (70 μm). Contrast images are obtained for both GE Pristina and Hologic 3Dimensions (for the Hologic Selenia Dimensions equipment CEM was not available) by using similar x-ray spectra as used for standard DM to acquire the LE images, employing a copper (Cu) filter and high tube voltage (45–49 kV_p_) to stretch the x-ray photon spectrum above the iodine absorption peak (33.2 keV) and acquire the HE image. Regarding the AEC modes, the Hologic systems have a unique AEC mode (“AutoFilter”) for each modality, while the GE Pristina has three AEC modes for DM (“AOP/STD,” “AOP/STD + ,” “AOP/DOSE”), two AEC modes for DBT (“AOP/STD,” “AOP/STD + ”), and one AEC mode for CEM (“AOP/STD”). AOP stands for “automated optimization of parameters,” while STD indicates the “standard” AEC mode for GE.

### Design of experiment

A variable-thickness phantom was created using stacked semicircular slabs of polymethyl methacrylate (PMMA), each 5-mm and 10-mm thick. The total thickness of the phantom varied from 20 to 70 mm, with intervals of 5 mm. In addition, an aluminum square of 15 × 15 mm^2^ surface and 0.2-mm thickness was placed on top of the initial 20-mm phantom to generate a signal difference in the resulting images. All other thicknesses were obtained by progressively overlapping PMMA slabs. A total of 363 studies were acquired among the available AEC modes for each phantom thickness and imaging modality on the three mammography units, comprising three sets of studies for each case. These studies included 165 DM studies (GE: 11 phantoms × 3 images × 3 AEC modes = 99; Hologic: 11 phantoms × 3 images × 1 AEC mode = 33 × 2 systems = 66), 132 DBT studies (GE: 11 phantoms × 3 series × 2 AEC modes = 66; Hologic: 11 phantoms × 3 images × 1 AEC mode = 33 × 2 systems =), and 66 CEM studies (11 phantoms × 3 series × 1 AEC mode = 33 for both manufacturers).

The technical factors (A/F, tube voltage, exposure) selected by the AEC were extracted from the image metadata (as Digital Imaging and Communications in Medicine [DICOM] tags), as well as the dose at the breast entrance and the calculated organ dose. However, because the organ dose can be calculated by different manufacturers under different assumptions, in this study mean glandular dose (MGD) was recalculated according to the model developed by Dance and colleagues [11–14] and used as the radiation dose metric.

**Fig. 1:**
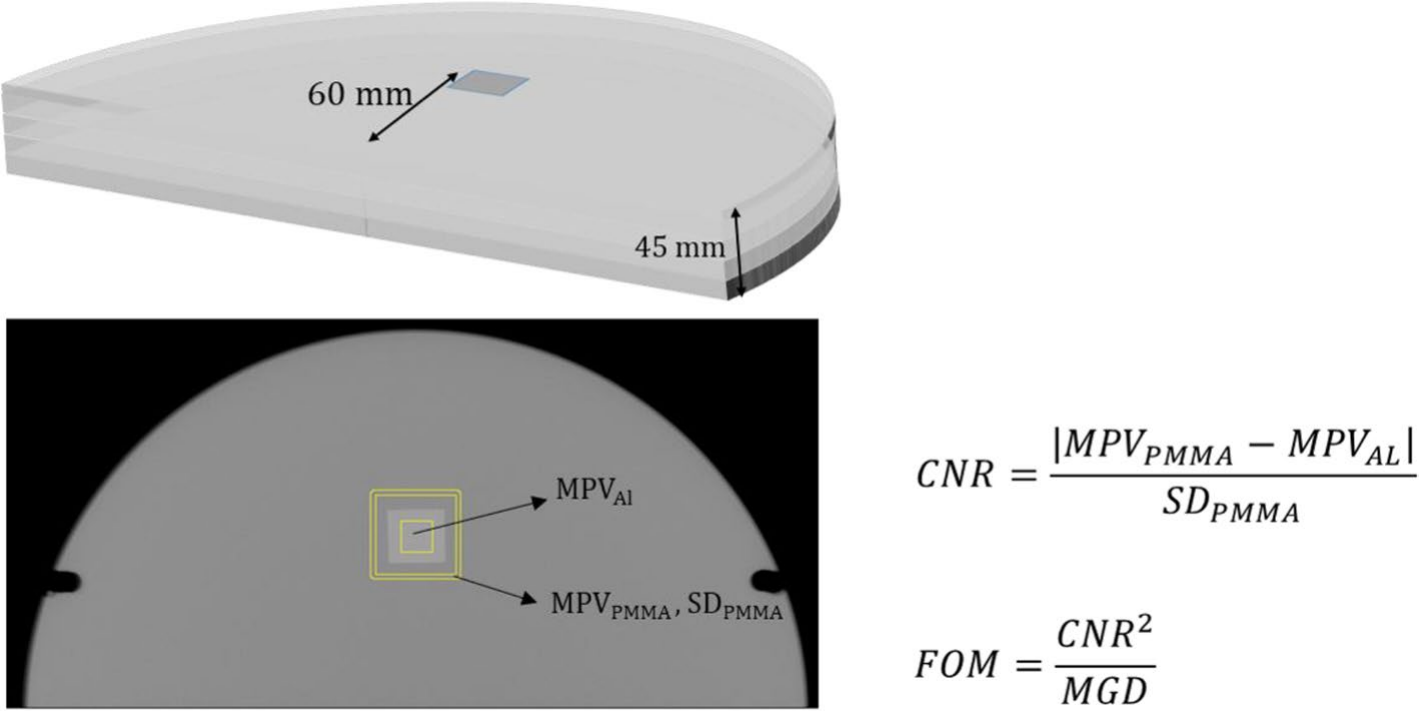
Experimental setup for the study demonstrating a representative case with 45-mm polymethyl methacrylate thickness. The illustration depicts the phantom structure, the position of the 0.2 mm Al square, designated regions of interest (ROIs) for signal measurement (mean pixel value, MPV), and noise measurement (standard deviation, SD) within and around the aluminum detail, and formulas for calculating contrast-to-noise ratio (CNR), and figure-of-merit (FOM)

IQ is a complex concept that includes multiple parameters, each associated with different factors such as contrast resolution, spatial resolution, and noise, which affect IQ. Systems for AEC are usually designed and calibrated using very reproducible metrics obtainable from simple phantoms. In this experiment, “DICOM for processing” images were used to measure the mean pixel value within the aluminum, Al, square (MPV_Al_) as well as the MPV_PMMA_ and standard deviation (SD_PMMA_) of the surrounding PMMA background, and to calculate the contrast-to-noise ratio (CNR) as a surrogate for IQ, and a figure-of-merit (FOM) calculated as CNR^2^/MGD. Figure 1 shows the phantom structure for a thickness of 45 mm, one of the images resulting from a DM-mode acquisition with the regions of interest (ROIs) within the Al detail and a square band surrounding the detail to obtain the MPV and SD values, and the formulas used to calculate the four IQ metrics.

### Statistical analysis

The mean values of the technical factors extracted from the DICOM image header, as well as the mean values of the calculated MGD, CNR, and FOM values, were computed on the three-sample series for each system, modality, AEC mode, and phantom thickness. Given the limited number of repeated images, measurement errors were calculated as maximum half-dispersion. The relative error of exposure, MGD, and CNR determined by dividing the maximum half-dispersion by the mean value was used to AEC the variability for each modality.

The distributions of quantitative technical factors (tube voltage and exposure) and those of calculated MGD, CNR, and FOM were analyzed by imaging modality, grouping any phantom thickness, system, and AEC mode. Since none of these data were normally distributed, median values and interquartile range (IQR) were used as estimators of each distribution. For CEM, median values and IQRs were provided separately for LE and HE images.

Variations of technical factors and derived MGD, CNR, and FOM were analyzed by imaging modality as a function of PMMA thickness for all available systems and AEC modes. Mean values from the three repeated acquisitions were used as data points and maximum half-dispersion represented as error bars. For DBT, total exposure and total MGD were used, representing the cumulative values required for all DBT projections. Data measured from images such as MPV_Al_, MPV_PMMA_, and SD_PMMA_ used to calculate CNR were obtained exclusively from the DBT projection at 0°; therefore, the FOM for DBT was calculated by dividing the CNR of the 0° projection by the square root of the MGD per projection obtained from the total MGD divided by the number of DBT projections. For CEM, all variables were analyzed separately for LE and HE images, with the exception of total MGD which was calculated as the sum of MGD values associated with LE and HE images.

Finally, a comparison of modality pairs (DBT *versus* DM and CEM *versus* DM) was performed by analyzing the MGD ratios calculated by dividing the MGD with DBT and with CEM for each phantom thickness and system by the corresponding MGD value with DM, and the CNR ratios obtained doing the same with the CNR. For the GE system, having more than one AEC mode available with DM and DBT, the AOP/STD mode was chosen for ratio calculation.

ImageJ2 [15] was used to measure the MPV and SD values from the DICOM imaged for CNR calculation.

Statistical analyses were performed by OriginPro 2020b (OriginLab Corporation, Northampton, MA, USA).

## Results

Table 2 presents the median and the interquartile range values for the resulting tube voltage, exposure, MGD, CNR, and FOM, distinguished by imaging modality. These values were derived from the full distributions for each modality, which include data from all phantom thickness, systems, and AEC modes. For CEM, median values and interquartile ranges were provided separately for LE and HE images.

**Table 2:** Median values and interquartile range for tube voltage, exposure, MGD, CNR, and FOM for DM DBT and CEM

	Median (interquartile range)		
Parameter	DM	DBT	CEM
Tube voltage (kV_p_)	31.0 (26.0–34.0)	31.5 (27.5–34.0)	LE = 29.5 (26.0–34.0) HE = 49.0 (45.0–49.0)
Exposure (mAs)	51.7 (30.2–94.9)	44.7 (32.7–59.2)	LE = 61.0 (33.5–134.6) HE = 102.8 (73.3–106.6)
MGD (mGy)	1.135 (0.772–1.668)	1.257 (0.971–1.863)	LE = 1.280 (0.937–1.878) HE = 0.630 (0.397–0.713)
CNR	14.2 (7.8–20.2)	4.91 (2.58–7.20)^a^	LE = 11.9 (8.0–18.2) HE = 5.2 (3.6–9.2)
FOM	141.5 (73.9–388.5)	150.4 (73.6–409.8)^b^	LE = 114.0 (47.5–268.3) HE = 63.0 (24.0–119.9)

These values were derived from the full distributions for each modality, which include data from all phantom thickness, systems, and AEC modes. For CEM, median values and IQRs are provided separately for LE and HE images

*CEM* Contrast-enhanced mammography, *CNR* Contrast-to-noise ratio, *DBT* Digital breast tomosynthesis, *DM* Digital mammography, FOM Figure-of-merit, *IQR* Interquartile range, *HE* High-energy image, *LE* Low-energy image, *MGD* Mean glandular dose

^a^The CNR in DBT was calculated only for the 0° projection

^b^FOM in DBT was calculated considering the CNR of the 0° projection and the MGD for a single projection

The median MGD values and IQRs were 1.135 mGy (0.772–1.668) in DM, 1.257 mGy (0.971–1.863) in DBT, 1.280 mGy (0.937–1.878) in LE-CEM, and 0.630 mGy (0.397–0.713) in HE-CEM. The CNR medians were 14.2 (7.8–20.2) for DM, 4.91 (2.58–7.20) for a single projection in DBT, 11.9 (8.0–18.2) for LE-CEM, and 5.2 (3.6–9.2) for HE-CEM. The variability of each parameter (tube voltage, exposure, MGD, and CNR) is shown in Table 3 for all three mammography systems, modalities, and AEC modes for each modality.

**Table 3:** Automatic exposure control repeatability: maximum relative error for tube voltage (kV_p_), exposure (mAs), calculated MGD and CNR obtained from measurements on the phantom images

Modality	GE Pristina AOP/STD	GE Pristina AOP/STD +	GE Pristina AOP/DOSE	Hologic Dimensions AutoFilter	Hologic 3Dimensions AutoFilter
DM	kV_p_ 0.00% mAs 2.25% MGD 2.27% CNR 1.77%	kV_p_ 0.00% mAs 1.60% MGD 1.51% CNR 1.49%	kV_p_ 0.00% mAs 1.92% MGD 1.74% CNR 3.35%	kV_p_ 0.00% mAs 2.14% MGD 2.06% CNR 1.28%	kV_p_ 0.00% mAs 2.46% MGD 2.39% CNR 1.07%
DBT	kV_p_ 0.00% mAs 1.86% MGD 2.22% CNR 2.56%	kV_p_ 0.00% mAs 1.37% MGD 1.75% CNR 1.68%	−	kV_p_ 0.00% mAs 1.36% MGD 0.50% CNR 2.32%	kV_p_ 0.00% mAs 1.30% MGD 1.35% CNR 1.41%
CEM					
LE	kV_p_ 0.00% mAs 1.17% MGD 1.20% CNR 1.20%	−	−	−	kV_p_ 0.00% mAs 3.03% MGD 3.14% CNR 2.25%
HE	kV_p_ 0.00% mAs 1.30% MGD 1.15% CNR 1.79%				kV_p_ 0.00% mAs 3.57% MGD 3.74% CNR 2.94%

The reported error values represent the highest variation observed across the entire range of phantom thicknesses for all three mammography systems and their respective modalities, along with the automatic exposure modes available for each modality. *CEM* Contrast-enhanced mammography, *CNR* Contrast-to-noise ratio, *DBT* Digital breast tomosynthesis, *DM* Digital mammography, *MGD* Mean glandular dose

The AECs of the three systems were found to be perfectly repeatable for the selection of anode/filter combination and tube voltage for all imaging modalities and phantom thicknesses. All AEC modes were highly repeatable in all modalities (DM, DBT, and CEM) with all variations less than 5%.

### AEC in DM

**Fig. 2:**
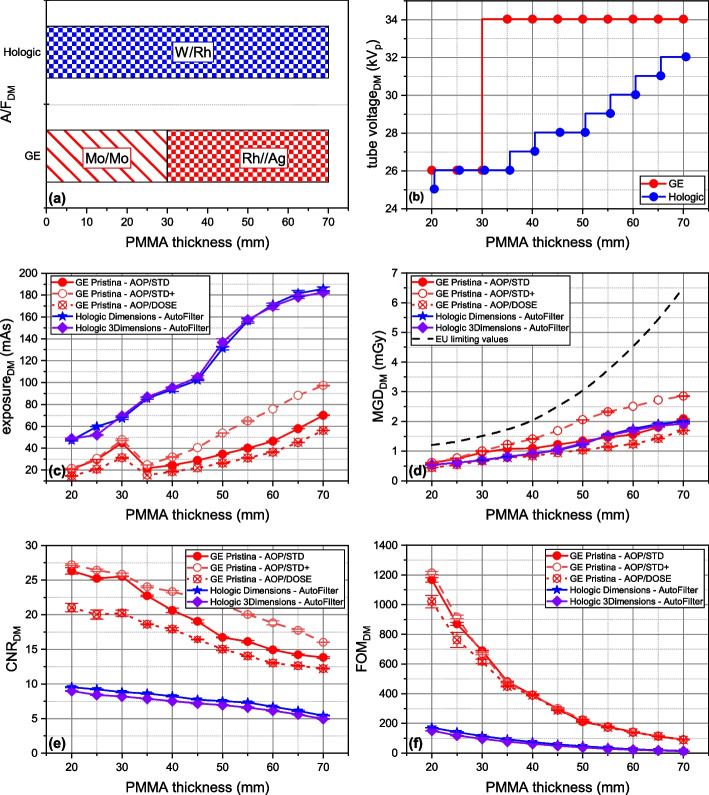
Automatic exposure control in digital mammography: behavior of technical factors, radiation dose, and image quality *versus* polymethyl methacrylate thickness. **a** Anode/filter combination. **b** Tube voltage (kV_p_). **c** Exposure (mAs). **d** Mean glandular dose, MGD (mGy). **e** Contrast-to-noise ratio (CNR). **f** Figure-of-merit (FOM)

Figure 2 provides a comprehensive overview of AEC behavior in the three systems compared for modality, including all available AEC modes. The panel includes (a) A/F combination, (b) tube voltage, (c) exposure, (d) MGD, (e) CNR, and (f) FOM.

Regarding the GE Pristina AEC, Fig. 2a, b shows a binary mode of operation: Mo/Mo@26kV_p_ for object < 35 mm PMMA-equivalent thickness and Rh/Ag@34kV_p_ for ≥ 35 mm thickness. X-ray spectra remained constant across the three AEC modes, with exposure (mAs) being incrementally adjusted for increased absorption with object thickness. Exposure was the only varying technical factor among the three AEC modes. In AOP/STD + mode, exposure was systematically increased compared to AOP/STD, while in AOP/DOSE mode, it was reduced.

In contrast, AutoFilter mode of both Hologic systems increased both tube voltage and exposure with phantom absorption. Within the phantom thickness range of 20–70 mm PMMA, only W/Rh anode/filter combination was chosen,^1^ and AEC behavior was similar between the two systems, selecting the same kV_p_ values, with small mAs differences (< 5%). Compared to the GE AOP/STD, AOP/STD + increased mean MGD by 32.5%, while AOP/DOSE decreased it by 24.0% on average. On average, the MGD of Hologic systems was 9.5% lower than GE AOP/STD mode. Overall, the average MGD was 1.26 mGy with 22.4% variability across systems and AEC modes, always staying below the EUREF dose limits [16].

Concerning the IQ metric, CNR (Fig. 2e) decreased with increasing object thickness, with distinct values for the three GE AEC modes due to exposure variations. AOP/STD + and AOP/DOSE *versus* AOP/STD showed a mean CNR gain of + 15.1% and a loss of -14.9%, respectively. CNR was consistently higher for GE (mean CNR 19.36) than for Hologic (mean CNR 7.42), with a mean increase of 61.7%. Overlapping FOMs for the three AEC modes of the GE system and for the two Hologic systems indicates that this FOM was also used by manufacturers for AEC optimization.

### AEC in DBT

**Fig. 3:**
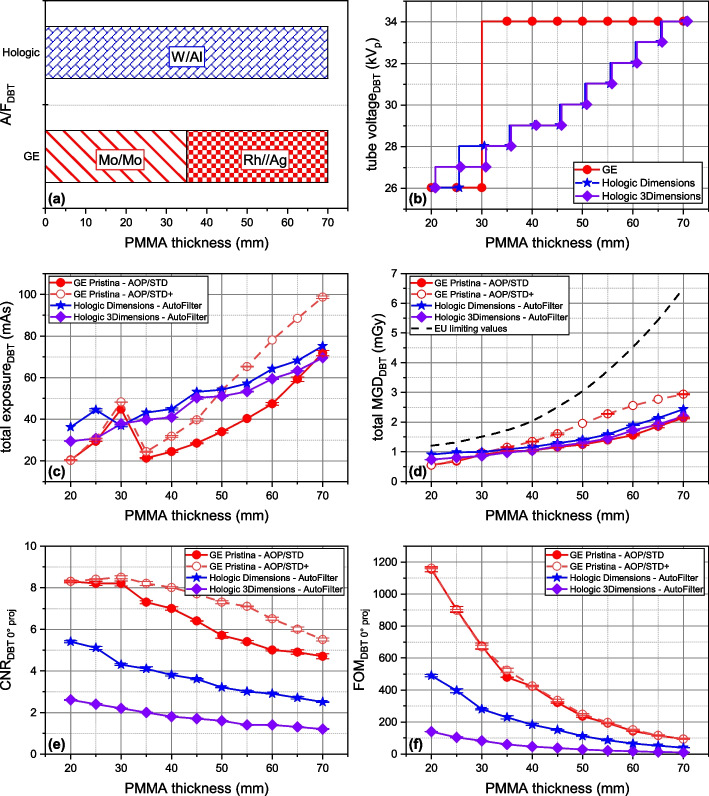
Automatic exposure control in digital breast tomosynthesis: behavior of technical factors, radiation dose, and image quality *versus* polymethyl methacrylate thickness. **a** Anode/filter combination. **b** Tube voltage (kV_p_). **c** Total exposure (mAs). **d** Total mean glandular dose, MGD (mGy). **e** Contrast-to-noise ratio (CNR) of the 0° projection. **f** Figure-of-merit (FOM) considering only the 0° projection

Unlike DM in which there are three AEC modes, the GE Pristina equipment uses two AEC modes in DBT, while the two Hologic systems maintain their single mode. Figure 3 provides a complete picture of AEC operation in DBT, with the dose-related parameters, specifically exposure and MGD, representing the cumulative sum of all DBT projections (9 for the GE system and 15 for the two Hologic systems). In contrast, CNR and FOM parameters correspond to a single projection (at 0°).

The GE Pristina maintained a binary x-ray beam spectrum pattern, switching from Mo/Mo@26kV_p_ for < 35 mm to Rh/Ag@34kV_p_ for objects ≥ 35 mm. Only the exposure value increased with object thickness. In contrast, the Hologic systems used a single W/Al anode/filter combination, adjusting both tube voltage and exposure based on object thickness. AOP/STD + operated at a mean MGD 33.4% higher than AOP/STD. On average, MGD of Hologic systems in DBT was 13.85% higher than GE AOP/STD mode. Overall, the mean total MGD in DBT was 1.41 mGy with 17.2% variability across systems and AEC modes, still below the EUREF dose limits [16].

In DBT, CNR (Fig. 3e) showed differences from mammography. Single-projection CNR in DBT was lower than in DM for all systems, but the mean CNR for the GE system was consistently higher than Hologic. On average, GE CNR (mean CNR 6.9) was 47.5% higher than Hologic Dimensions (mean CNR 3.7) and 74.7% higher than Hologic 3Dimensions (mean CNR 1.8). AOP/STD + exhibited a mean CNR gain of + 16.5% *versus* AOP/STD. Of note, CNRs and FOM overlap for the two Hologic in mammography, but diverged in DBT due to different detector binning.

### AEC in CEM

**Fig. 4:**
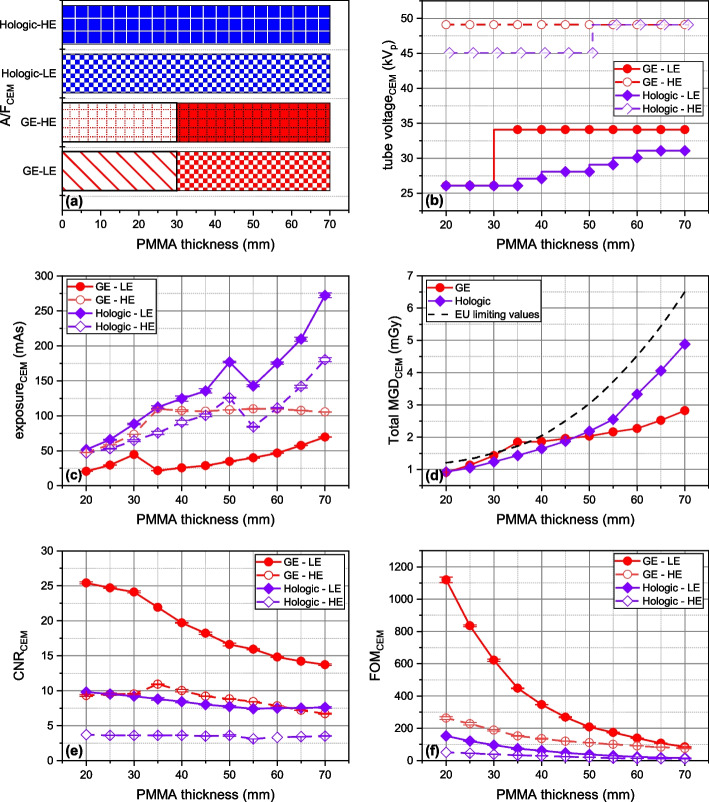
Automatic exposure control in contrast-enhanced mammography acquisition: behavior in terms of technical factors, radiation dose *versus* polymethyl methacrylate thickness. **a** Anode/filter combination. **b** Tube voltage (kV_p_). **c** Exposure (mAs). **d** Mean glandular dose, MGD (mGy). **e** Contrast-to-noise ratio (CNR). **f** Figure-of-merit (FOM). *HE* High-energy, *LE* Low-energy

GE Pristina and Hologic 3Dimensions, both equipped for contrast-enhanced mammography, use a single AEC mode in CEM. The behavior of AEC for the CEM mode is depicted in Fig. 4. All parameters are represented separately for LE and HE images, except for MGD reported as a total value.

For the LE images, the GE CEM system employed technical factors and MGDs identical to those used in mammography with AOP/STD AEC mode. On the other hand, the Hologic system with AutoFilter AEC demonstrated different choices in DM and CEM, operating with a higher MGD to acquire LE-CEM than standard mammography. In both systems, HE images were obtained by replacing the filter used for LE image acquisition with a Cu filter. The GE system uses a single tube voltage value of 49 kV_p_, while the Hologic system uses two separate values (45 and 49 kV_p_) for HE image acquisition. The mean MGDs for LE images were 1.285 mGy for GE and 1.683 mGy for Hologic, resulting in a mean difference of 0.398 mGy, indicating a Hologic increase of 30.9%. The mean MGD was comparable for both manufacturers in HE images (GE = 0.611 mGy, Hologic = 0.596 mGy), with a small 2.4% advantage for Hologic. Considering the total MGD, Hologic showed a mean increase of 20.2% over GE (GE = 1.896 mGy, Hologic = 2.279 mGy; difference 0.383 mGy). Combining data from both CEM systems and all phantom thicknesses, the mean MGD was 2.088 mGy with 10.4% variability.

The GE system showed higher CNR and FOM in both LE and HE images compared to Hologic. CNR was 58.3% higher for the GE system.

### Intermodality comparison

**Fig. 5:**
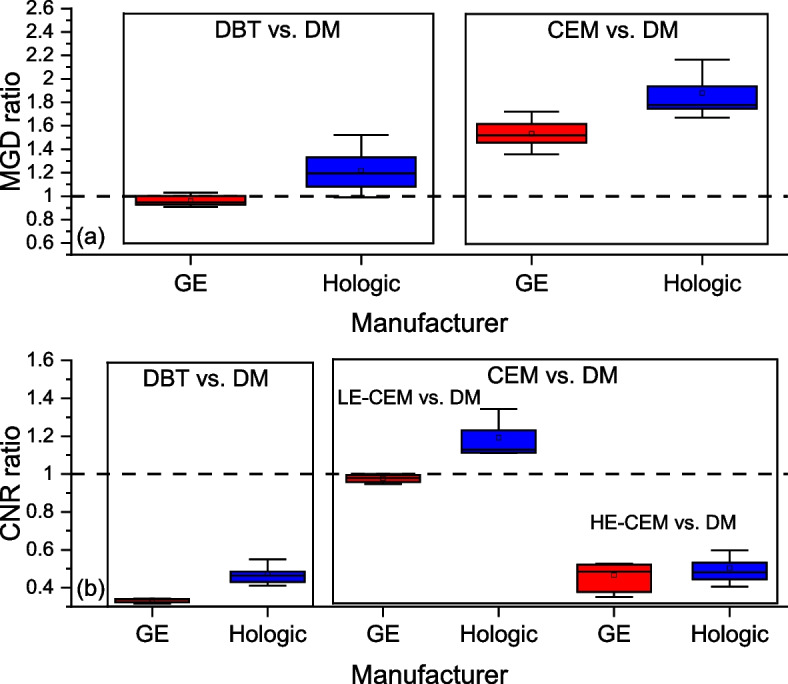
Box plots of (**a**) mean glandular dose (MGD) ratios and (**b**) contrast-to-noise (CNR) ratios between digital breast tomosynthesis (DBT) and digital mammography (DM) and between contrast-enhanced mammography (CEM) acquisitions and DM for the entire phantom thickness range. A ratio equal to 1 indicates the same MGD or CNR in DM and in the compared modality

Figure 5 shows the box plots of MGD and CNR ratios between DBT and DM and between CEM and DM for the entire phantom thickness range.

The mean MGD ratio comparing DBT to DM was 0.96 for GE and 1.22 with Hologic, while the ratio between CEM and DM was 1.53 for GE and 1.88 for Hologic. The mean CNR ratio between DBT and DM was 0.33 for GE and 0.47 for Hologic, whereas it was 0.98 and 1.19 between LE-CEM and DM and 0.47 and 0.50 between HE-CEM and DM.

## Discussion

Examination of the AEC behavior of three mammography systems for DM, DBT, and CEM reveals nuanced variations impacting radiation dose and IQ. The observed variations in AEC behavior are attributed to differences in the components, such as x-ray tube and image detector characteristics employed by various manufacturers, but also reflect the different “philosophies” adopted in the design of AEC systems. In the GE system, the goal was to maintain consistency by employing identical technical factors, including anode/filter combination, tube voltage, and exposure, in all imaging modalities for the same AEC option. In addition, GE added versatility providing radiographers with three AEC modes for DM and two for DBT. Instead, Hologic took a different approach, providing a single AEC modality applicable to all modalities, including DM, DBT, and CEM. However, unlike the GE system, Hologic opted for separate technical factors and operated with increasing dose levels, moving from DM to DBT to CEM.

In this study, the mean MGDs computed by averaging across systems for each modality were 1.26 mGy with DM, 1.41 mGy with DBT, and 2.09 mGy with CEM, with a variability of 22.4%, 17.2%, and 10.4%, respectively. Given the considerable variability across systems and AEC modes, it is recommended to be cautious in generalizing conclusions about dose level for specific modalities or differences in dose between modalities, especially when based on results derived from a single piece of equipment. Finally, it is important to note that the mean MGD differences between modalities observed in this phantom-based study may not be directly related to clinical dose results [17–21]. In fact, the uniform distribution of phantoms across the entire thickness range (20–70 mm PMMA) contrasts with the specific distributions of breast thicknesses in clinical populations. Consequently, MGD differences between modalities might manifest differently when applied to real-world scenarios with different breast thickness distributions.

However, when evaluating the AEC performance, it is clear that while MGD allows for comparison across different manufacturers and modalities, the same is not true for simple IQ metrics such as CNR. In this study, the GE system showed a much higher CNR than Hologic in all modalities. Although CNR is recognized as a valid IQ indicator, particularly for quantifying the “visibility” of low-contrast objects in the image [22, 23], it cannot capture other crucial aspects of IQ, such as spatial resolution, and may not be consistently correlated directly with clinical relevance. In terms of AEC operation, maintaining a consistent CNR level for various object thicknesses proves to be more significant than maximizing its absolute value. In fact, the general principle is that as breast absorption increases, the AEC should be able to compensate for the reduction in radiation dose reaching the imaging detector by adjusting the choice of technical factors to effectively “preserve” IQ [24]. Considering a reference thickness such as 45 or 50 mm PMMA, CNR reduction operating in AEC mode for thicker objects was within -30% for 70-mm PMMA (equivalent to 90 mm breast) for all systems and modalities. This CNR reduction of less than 30% from a reference thickness is considered acceptable by some quality control protocols [15, 16].

The results of this study are in line with the technical measures reported by the National Health System England for each manufacturer and imaging modality [25]. It is clear that the use of a FOM derived from CNR and MGD [26, 27] represents only one of several possible options for optimizing the automatic exposure control [7, 28]. It is a compromise choice, ensuring that IQ is preserved to a reasonable extent as breast thickness increases, while keeping the radiation dose as low as possible and extending the lifetime of equipment components, such as the x-ray tube and detector. Salvagnini et al. [28] showed that to keep CNR constant, an increase in MGD between 15 and 73% is required for PMMA thicknesses between 40 and 70 mm.

In this work, CNR was obtained from “DICOM for processing” images for all modalities, including DBT; the same choice was done by the EUREF protocol [16], while other quality control protocols as that applied for the ECOG-ACRIN EA1151 TMIST trial measure the CNR as reproducibility test from the reconstructed “DICOM for presentation” images [29]. The idea behind using “for processing” images is that any gain/loss of IQ in such images will result in a gain/loss of IQ in the processed (DM) or reconstructed (DBT) or recombined (CEM) images.

Although this study provides a valid comparison of AEC operations for a limited number of equipment models from two manufacturers, it is important to recognize its inherent limitations. In particular, the study does not include the full spectrum of existing mammography systems, as numerous other systems with different functionalities were not included in the analysis. The primary objective of this research was not to capture the entire landscape of AECs in all mammography systems, but rather to highlight differences in automatic exposure controls between specific models, offering insights into their impact on both radiation dose and IQ under controlled conditions. Another limitation is the use of CNR measured on “for processing” images as the only IQ metric when comparing AEC comparison, with potential variations using different metrics or using “for presentation” images. Assessing the behavior of AECs by considering different metrics was outside the scope of the study.

In conclusion, the results of the study emphasized the central role of AEC devices, showing their distinct operation in different mammography systems and modalities. The observed variations highlight the impact of differences in technical components and manufacturers’ strategies on system design. These variations have potential implications for both radiation dose and IQ in clinical settings, underscoring the need to avoid broad generalizations from results obtained from a single source of equipment.

## Data Availability

The datasets analyzed during the current study are available in the Zenodo repository, https://zenodo.org/records/10159204.

## Abbreviations

A/F: Anode/filter combination
AEC: Automatic exposure control
AOP: Automatic optimization of parameters
CEM: Contrast-enhanced mammography
CNR: Contrast-to-noise ratio
DBT: Digital breast tomosynthesis
DICOM: Digital Imaging and Communications in Medicine
DM: Digital mammography
FOM: Figure-of-merit
FPD: Flat panel detector
HE: High-energy
IQ: Image quality
IQR: Interquartile range
LE: Low-energy
MGD: Mean glandular dose
MPV: Mean pixel value
PMMA: Polymethyl methacrylate
SD: Standard deviation
STD: Standard mode

## Footnotes

1 Switching to the W/Ag combination requires objects thicker than 70 mm.
